# Abortion care in a pandemic: an analysis of the number and social profile of people requesting and receiving abortion care during the first COVID-19 lockdown (March 16 to June 14, 2020) in Flanders, Belgium

**DOI:** 10.1186/s13690-021-00665-6

**Published:** 2021-08-04

**Authors:** Leen De Kort, Jonas Wood, Edwin Wouters, Sarah Van de Velde

**Affiliations:** Centre for Population, Family and Health, Department of Sociology, University of Antwerp, Sint-Jacobstraat 2-4, 2000 Antwerp, Belgium

**Keywords:** COVID-19, Abortion, Belgium, Reproductive health, Healthcare services

## Abstract

**Background:**

The COVID-19 pandemic and the national COVID-19 measures might have increased potential barriers to abortion care and created new ones, especially for vulnerable groups. This study documents the impact of the pandemic and the lockdown measures on the profile of people going through the abortion process.

**Methods:**

Using anonymized patient records from a Belgian abortion centre, we first compared the number of abortion requests and procedures during the first COVID-19 lockdown with the same months in the five preceding years. Next, we analysed the social profile of people requesting an abortion in those two time periods and looked at the number of long-acting reversible contraceptive devices (LARC) placed after curettage.

**Results:**

The abortion centre saw a drop in the number of abortion requests during the lockdown. This difference was more pronounced for people in paid employment and people using (modern) contraception. People were also more likely to request an abortion earlier in their pregnancy. The drop in abortion procedures and LARC’s placed after curettage was proportionate to the drop in abortion requests and did not differ according to clients’ characteristics.

**Conclusion:**

Questions arose concerning the potential selectivity with which COVID-19 influenced the need for abortion care and accessibility to services. Although there was a general drop in abortion requests and procedures during the first COVID-19 lockdown in the studied abortion centre, our results suggest that the profile of people requesting and receiving an abortion did only slightly change during the lockdown, and did not affect vulnerable groups visibly harder.

**Supplementary Information:**

The online version contains supplementary material available at 10.1186/s13690-021-00665-6.

## Background

For Belgium, governmental COVID-19 measures were most stringent during the first lockdown, which started on the 14th of March 2020. Schools, restaurants, and non-essential shops were closed, teleworking was mandated for “non-essential” occupations, and people were only permitted to leave the house for essential activities or limited outdoor exercise with close family members or one friend. The country also closed its borders. Measures were relaxed from the 10th of May on, and borders opened from the 15th of June. These measures significantly impacted the organization of Belgian primary health care (PHC) services, where quick adaptations in practice management and consultation strategies were necessary to guarantee safe practices [1–3].

Concerning abortion care, possible pre-existing difficulties such as arranging childcare, taking time off from work, and transport to the abortion centre [4–9] may have potentially been increased by both the pandemic itself and the protective measures installed by the Belgian government. Additionally, the COVID-19 pandemic may have resulted in new barriers due to, for example, conflicting media messages concerning the availability of PHC-services, fear of infection with COVID-19 when using public transport or visiting PHC-services, and fear of being sanctioned for traveling outside the house. Although Belgian abortion centres are located very central, the practical side of arranging abortion care might have been compromised by the COVID-19 measures; schools were closed, telework was mandated, and people were discouraged from making use of grandparents to look after children. In addition, secrecy might have been compromised by continually having family members around, and additionally, one was not able to use cultural/social activities as an alibi. Furthermore, telephone consultations (aimed at minimizing the number of contact moments between clients and staff) might have resulted in more communication difficulties [3]. By Belgian law, curettages require two visits to the abortion centre, while medical abortions require three visits to the abortion centre (see the ‘context’ section). Because of this additional in-person visit, some centres also stopped offering medical abortions during the lockdown, thus reducing the options for people in need of abortion care (see [3] for more information on this change in procedure in the abortion centre which is the focus of this study). Since abortion care could not be offered to people with COVID-19 symptoms, this may also have created additional barriers.

Additionally, the question arises whether the COVID-19 pandemic and lockdown measures affected all groups equally in terms of their progress through the various steps of the abortion service (abortion request, abortion procedure, and placing long-acting contraceptive devices (LARC)). Under normal circumstances, socioeconomically vulnerable people and people with a migration background were already more likely to experience barriers in accessing abortion care [10–13]. This risk may have been elevated due to higher COVID-19 infection risk in people living in urban and deprived areas [14, 15], and sudden income loss caused by lockdown measures [16].

Although there are many reasons to suspect that COVID-19 affected people in need of abortion care, to date, empirical research on this topic is scarce. Available research shows that people encountered more difficulties in obtaining timely sexual and reproductive health (SRH) care services [17], and that in countries with strict COVID-19 regulations, the demand for self-managed abortion increased [18]. Other research shows that solutions, including self-managed abortions through telemedicine, were well received by clients [19–21]. In Belgium, previous research found that the staff of the abortion centre was worried about the level of psychological support they were able to offer to vulnerable groups during the lockdown [3].

With the current study, we contribute to this body of empirical research by examining one of the bigger Flemish abortion centres and studying (1) whether COVID-19 had an impact on the number of people progressing through the various steps of the abortion service (abortion request, abortion procedure, and placing LARC), as well as (2) whether certain groups of people were affected more severely.

### Context

In 1990, Belgium partially depenalised abortion by making abortion on demand legal in the first 12 weeks of a pregnancy (after this period, abortion is possible only when the life of the mother is in danger or if serious abnormalities are found in the foetus). A report from an opinion poll conducted in 2017 showed that 75,4% of the participants agreed with bills proposing to also remove abortion from the criminal code [22], and in October 2018, this change of the law officially completed the depenalisation of abortion in Belgium.

When a client contacts one of the abortion centres, an appointment at the abortion centre will be offered within 10 days. During this first appointment a consultation with a social worker or psychologist, and a medical check-up take place. The second appointment in which the abortion will be performed, can be scheduled after a six-day waiting period. If the unwanted pregnancy is younger than 7 weeks, the client can choose between a curettage or a medical abortion (unless there are medical reasons to prefer one method over the other). After 7 weeks of conception, only a curettage is possible. The procedures take place in the abortion centre. This is also the case for the medical abortion, where both the ingestion of mifepristone (during the second appointment) and the ingestion of misoprostol (during an additional third appointment) need to happen under the supervision of an abortion centre staff member.

All people residing in Belgium have government-sponsored health insurance, which covers most costs of abortions (clients only pay 3.68 euro). For non-regularized migrants, abortion is seen as urgent medical help, which means that a refugee centre or the public social welfare office (‘openbaar centrum voor maatschappelijk welzijn’, or in short ‘OCMW’) can interfere in the costs.

## Methods

The current study was built on a collaboration with the network of Dutch speaking non-hospital-based abortion centres in Flanders (Belgium) where about 95,67% of the registered abortions on demand in Flanders occur [23]. We used their anonymized patient records for our analysis. This allowed us to use population data, hence ruling out sampling issues. Our study focused on one of the four abortion centres from this network, which is located in a central city and receives clients from the entire region. This centre implemented strict protective measures (e.g., mandatory telephone consultations, no medical abortions, no company allowed for the procedure, mask-wearing mandatory) against COVID-19 between March 16 and June 14, 2020 (hereafter referred to as the lockdown).

We used descriptive results to compare the number of abortion requests, abortions, curettages, and medical abortions in the lockdown period with the same period in the five preceding years (2015–2019), hereafter referred to as the reference period. Within these periods between 2015 and 2020, 4243 abortion requests were registered at the abortion centre, and thus included in the study.

We also analysed whether the social profile of people who requested an abortion differed significantly between the reference and lockdown period. To this end, we ran bivariate regressions with ‘COVID-19 lockdown’ as the independent variable (value ‘1’ for the lockdown) and the profile characteristics as dependent variables. Linear regressions were performed for the continuous variables (see Table 1), while multinomial logistic models were used for the categorical variables (see Table 2). For variables with missing information (employment status, educational level and contraceptive use), we created a separate ‘unknown’ category (see Additional file 1: Table B). Unfortunately, this category was rather large for the variable ‘educational degree’ (between 7.69 and 19.04% for the 2015–2019 period, and even 30.27% for the lockdown period in 2020). This needs to be kept in mind when interpreting the results related to this variable. When referring to types of contraception, permanent methods such as sterilisation, long-acting reversible contraception, hormonal contraception, barrier methods, and emergency contraception were seen as modern methods, whereas coïtus interruptus and natural family planning methods were not. To control for potential longer trend effects unrelated to the COVID-19 lockdown, we contrasted 2020 with 2019 only and ran the analysis with a linear calendar time covariate as sensitivity checks. As bivariate identifications of specific profile changes might also be caused by compositional changes in terms of other profile characteristics, we also carried out sensitivity models in which the other profile characteristics were used as covariates. Results of these analyses are only mentioned when relevant.

**Table 1:** Comparison between the reference period (2015–2019) and the first COVID-19 lockdown (2020) of profile characteristics of the clients from an abortion centre in a central city in Flanders (Belgium), based on bivariate linear regressions (*n* = 4243)

	Average 2015–2019	Average 2020	Regression coefficient
Model a1: Age	29.02	29.62	0.62*
Model a2: Number of children	1.13	1.24	0.11
Model a3: Number of previous abortions	0.66	0.72	0.07
Model a4: Number of miscarriages	0.22	0.27	0.05

*p < .05. **p < .01. ***p < .001

**Table 2:** Comparison between the reference period (2015–2019) and the first COVID-19 lockdown (2020) of profile characteristics of the clients from an abortion centre in a central city in Flanders (Belgium), based on bivariate (multinomial) logistic regressions (*n* = 4243)

		Average % 2015–2019	% 2020	Odds-ratio
Model b1: Migration background	No migration background (ref. cat.)	43.41	40.82	/
	Western- and Southern Europe	6.00	7.14	1.26
	Balkan/central Europe/western CIS and Caucasus	12.23	11.73	1.01
	Maghreb	11.03	11.39	1.09
	Turkey	1.62	1.87	1.25
	Middle East	2.55	4.59	1.93**
	Sub-Saharan Africa	12.01	11.90	1.06
	America and Oceania	5.40	6.97	1.39
	Asia	5.71	3.57	0.66
Model b2: Employment status	In paid employment (ref. cat.)	61.07	56.46	/
	Studying	6.89	7.82	1.22
	Not employed nor studying	22.47	27.21	1.31**
	Unknown	9.57	8.50	0.96
Model b3: Educational level	Tertiary education	22.04	17.35	1.01
	Non-tertiary education (ref. cat.)	62.60	48.64	/
	Unknown foreign education	2.68	3.74	1.78*
	Unknown	12.60	30.27	3.09***
Model b4: Marital status	Single/living alone (ref. cat.)	49.58	51.36	/
	Married	18.75	22.45	1.15
	Unmarried cohabitation	26.14	22.45	0.85
	Divorced/in process/widowed	4.94	3.74	0.59*
Model b5: Pregnancy term	0–8 weeks (ref. cat.)	58.02	62.41	/
	9–14 weeks	25.95	21.77	0.79*
	No abortion	16.03	15.82	0.92
Model b6: Contraceptive method	No modern method (ref. cat.)	50.63	63.78	/
	Modern method	49.37	36.22	0.58***
Model b7: Contraceptive use	No usage (ref. cat.)	45.53	57.14	/
	Irregular or inaccurate usage	30.57	26.02	0.68***
	Correct usage	17.93	12.76	0.57***
	Unknown	5.98	4.08	0.54**

*p < .05. **p < .01. ***p < .001

However, not all abortion requests result in actual abortions, and not everyone opts for LARC’s afterwards. The progression through this process might differ between people, depending on experiences with the first (in Belgium mandatory) counselling, and with barriers that might arise between the different stages. We examined whether the lockdown affected the progression to the various stages of obtaining abortion care (first consultation, abortion procedure, post-abortion contraceptive care), and whether this progression varied across different social profiles of people. In the first step, the dependent variable was having an abortion (value ‘1’) or not receiving an abortion at the abortion centre (value ‘0’) after requesting one. We ran a bivariate logistic regression with ‘COVID-19 lockdown’ as independent variable, and we estimated interactions between the COVID-lockdown and individual characteristics in a multivariate logistic regression. We excluded ‘term’ because this information is not available for those who did not have an abortion. Here again, we also estimated sensitivity models contrasting 2020 with 2019 or including a linear calendar time covariate. In a second step, we looked at people who had a curettage (*n* = 3096) and distinguished in the dependent variable between those who had a LARC placed after the procedure (value ‘1’) and those who did not (value ‘0’). Again, we ran a bivariate logistic regression with ‘COVID-19 lockdown’ as independent variable, and we estimated interactions between the COVID-lockdown and people’s characteristics in a multivariate logistic regression. We also contrasted 2020 with 2019 only, and ran the analysis with a linear calendar time covariate as sensitivity checks.

## Results

### Step 1: abortion requests

**Fig. 1:**
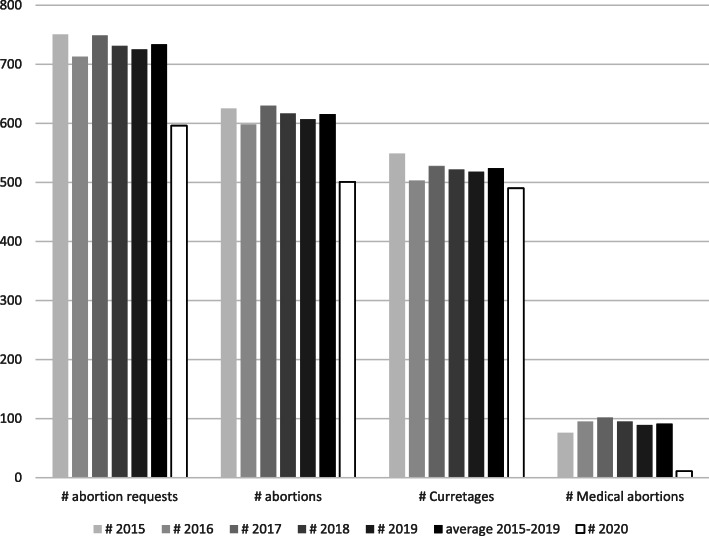
Number of abortion requests, abortions, and type of abortion in an abortion centre in a central city in Flanders (Belgium) during the first COVID-19 lockdown (2020) and the reference period (2015–2019)

There was a drop in both the number of abortion requests and actual abortions during the first lockdown compared to the previous years (see Fig. 1). On average, 733.8 abortions requests and 615.4 abortions took place every year during the reference period, while during the lockdown, only 596 requests and 501 abortions were registered. The drop in medical abortions was the largest (from on average 91.4 in the reference period to only 11 during the lockdown). However, also the number of curettages dropped (from on average 524 to 490 respectively).

The average age of a person requesting an abortion was significantly higher during the lockdown than in the reference period (see Table 1). However, as the descriptive statistics (see Additional file 1: Table A) show, the average age of a person requesting an abortion was slowly increasing over the past 5 years. It is therefore unlikely that COVID-19 caused this effect.

During the lockdown, it was more likely than in the reference period that people requesting an abortion had a Middle Eastern background (compared to people without a migration background) (see Table 2). However, when contrasting only with 2019, adding the calendar time covariate or other profile characteristics, the difference did not hold. The descriptive results of the employment category (see Additional file 1: Table B), show that during the lockdown, abortion requests dropped for all the categories, but to a greater extent in the ‘paid employment’-category. Our analyses confirmed that people requesting an abortion were more likely to be unemployed or still studying during the lockdown. Furthermore, the probability requesting an abortion when having an unknown foreign education was higher in the lockdown than during the reference period (compared to those who had no tertiary degree). During the lockdown, people requesting an abortion were less likely to be divorced or widowed than in the reference period (compared to being single or living alone). However, this difference did not remain significant when contrasting with only 2019 or adding the calendar time covariate. It was also more likely during the lockdown than in the reference period that people requested an abortion earlier in their pregnancy. This difference did not remain significant when contrasting 2019 with 2020 or when adding the calendar time covariate, but it did remain when adding the other characteristics as covariates. The descriptive results (see Additional file 1: Table B) show that although abortion requests dropped for all categories of this variable during the lockdown, the drop was larger for people who were 9 to 14 weeks along. Concerning contraceptive methods, people requesting an abortion were much less likely to use a modern contraception method during the lockdown than in the reference period and more likely not to use contraception overall.

### Step 2: from abortion requests to abortion procedures

When comparing the lockdown with the reference period, there was no significant difference between the percentages of people who effectively received an abortion at the abortion centre after requesting one (see Additional file 1: Table C for descriptive results). Neither did we find significant interactions between the COVID-19 lockdown and people’s profile characteristics for this transition.

### Step 3: from abortion procedures to placing LARC

Compared to the reference period, clients who received an abortion during the COVID-19 lockdown were not less or more likely to get LARC placed right after their abortion procedure (see Additional file 1: Table C for descriptive results). There were also no significant interactions between the COVID-19 lockdown and people’s profile characteristics in the transition from abortion procedure to having LARC placed.

## Discussion

Our study finds a substantial drop in the number of abortion requests during the first wave of the COVID-19 pandemic compared to the reference period. Although the government did not prohibit specific services and all abortion centres remained operational, health care facilities had to ensure sufficient capacity to treat COVID-19 patients and could therefore choose to suspend certain services. This caused conflicting messages (both in media and in personal networks) concerning the availability of PHC-services. Combined with the fear of contracting the virus on the way to or in PHC-facilities, people might have delayed seeking SRH-care. In Belgium, available qualitative research found that people indeed feared that abortion services were suspended and reacted relieved upon finding out that this was not the case [3]. A possible explanation for the drop in abortion requests is that people possibly engaged less in sexual risk behaviour during the lockdown [24, 25], and therefore were confronted less with unexpected pregnancies. However, research also found that in countries with strict COVID-19 measures, the demand for self-managed abortions increased [18].

The drop in abortion requests was more pronounced for certain groups. Compared to the reference period, a smaller proportion of the people who requested an abortion was in paid employment and used (modern) contraception during the lockdown. This finding does not seem to support the concern that specifically people in vulnerable positions had difficulties accessing the abortion centre. The observed drop in (modern) contraception use amongst people requesting an abortion during the COVID-19 lockdown could possibly be related to increased barriers concerning accessing contraceptive care in general during this period. People were also more likely to request an abortion earlier in their pregnancy. This result is in line with previous research in Belgium where the abortion clinic’s staff had the impression that during the lockdown, people inquired about abortion earlier in their pregnancy [3].

There was also a drop in actual abortions. The centre that was studied decided only to perform curettages during the first lockdown (because in Belgium, medical abortions require more in-person visits). However, clients who did not want to opt for a curettage were referred to a small independent institution at a nearby hospital, or could contact another abortion centre. Although we do not have information about who initially considered a medical abortion, we did see a small increase in the number of medical abortions in the referral hospital during the lockdown (from on average 69,8 in the reference period to 86 in the lockdown), whilst the number of curettages performed here remained stable throughout that period. This increase is however not proportionate to the decrease in medical abortions seen in the abortion centre. Additionally, the aggregated numbers of the Flemish network of abortion centres also show a decline in both abortion requests and abortions, although two of the four centres in this network did continue to provide medical abortions. This is still the case when adding on the numbers of the referral institution for medical abortions. Knowing that medical abortions require three in-person visits in Belgium (one psychosocial consultation, one visit to take Mifepristone, and a last visit to take Misoprostol and stay for an observation period), the choice not to offer them in order to limit in-person visits during the COVID-19 lockdown seems reasonable. However, within the international context, where tele-medicine and self-managed medical abortions are increasingly used to give people more options to safely terminate an unwanted pregnancy, this choice feels unfortunate. Nevertheless, the choice was re-evaluated after the first lockdown, and COVID-19 was used as an opportunity to try out partially self-managed medical abortions at home (see [3] for more information on this issue). Evaluations of this new practice could influence future policies concerning safe, medical abortions at home.

Since the abortion service consists of several stages (requesting an abortion, having the first consultation, having the actual abortion, and possibly having LARC placed after the procedure), we also wondered whether barriers arose between these different stages due to COVID-19. However, in our analysis, we did not find proof for this concern. Furthermore, there was no difference between the ratio of requests and actual abortions when comparing the lockdown with the reference period. Therefore, it seems that for those who found their way to the abortion centre, the ability of the centre to help their clients did not differ from before the COVID-19 lockdown.

Our study has several limitations. First, while we can suggest possible explanations for the observed drop in abortion requests, their power is limited because we do not have data concerning (unwanted) pregnancies in the reference and the lockdown period, or about unsafe abortions. Second, although the Flemish abortion centres are organised as a network, they function independently. While they operate under the same law and generally follow the same procedures, every centre reacted differently to the COVID-19 crisis. Therefore, our findings cannot be extrapolated to the other Belgian abortion centres, but need to be seen as an assessment of the local situation. Finally, the variable educational degree had a high percentage of missing cases overall, and this problem increased during the lockdown. This is unfortunate because educational status is usually seen as an important indicator in one’s social profile. Our results concerning this characteristic thus need to be read with caution.

## Conclusion

The COVID-19 pandemic and measures impacted the organisation of abortion services worldwide. Previous research confirmed that this was also the case in the Flemish part of Belgium, where in their attempt to safeguard abortion services, the abortion centres rapidly adapted their procedures [3]. Combined with the COVID-19 disease itself and the measures installed by the government, this raised the concern that barriers to abortion care increased. Our research aimed to help the abortion service specifically and abortion policies in Belgium in general to evaluate this situation. Although we could not directly study those who did not find their way to the abortion services due to COVID-19 related barriers, our research offers an indirect assessment of the accessibility of one of the mayor abortion services by comparing the numbers and the social profile of those who requested and received an abortion at the centre before and during the lockdown. For this assessment, we used population data, hence ruling out sampling issues. A similar assessment in the other Flemish abortion centres would be beneficial. Furthermore, it would remain very interesting for future research to gather people who experienced an unwanted or unexpected pregnancy during the lockdown and interview them concerning their experiences with SRH-services in this exceptional period.

## Supplementary Information

**Additional file 1:** **Table A:** Descriptive results of the continuous profile characteristics of the clients of an abortion centre in a central city in Flanders (Belgium), for the first COVID-19 lockdown (2020) and each year in the reference period (2015-2019), *n* = 4243. **Table B:** Descriptive results of the categorical profile characteristics of the clients of an abortion centre in a central city in Flanders (Belgium), for the first COVID-19 lockdown (2020) and each year in the reference period (2015-2019), *n* = 4243. **Table C:** Descriptive results of the amount of actual abortions and placing of LARC's, performed for the clients of an abortion centre in a central city in Flanders (Belgium), for the first COVID-19 lockdown (2020) and each year in the reference period (2015-2019).

## Data Availability

For this study, we made use of anonymized patient records. This data is not publicly available, but can be used for research after approval from the services who collect the data, and after obtaining clearance from an ethical committee.

## Abbreviations

COVID-19: 2019 Novel coronavirus
LARC: Long-acting reversible contraception
PHC: Primary health care
SRH: Sexual and reprodctive health

## References

[1] Verhoeven V, Tsakitzidis G, Philips H, van Royen P (2020). Impact of the COVID-19 pandemic on the core functions of primary care: will the cure be worse than the disease? A qualitative interview study in Flemish GPs. BMJ Open.

[2] Nationaal Intermutualistisch College (NIC) Intermutualistische enquete raadplegingen op afstand. 2020 Consulted on the 5th of February 2021. Retrieved from: https://www.riziv.fgov.be/nl/themas/kost-terugbetaling/door-ziekenfonds/Paginas/zorg-afstand-wat-leren-eerste-ervaringen.aspx

[3] De Kort L, Wouters E, Van de Velde S (2021). Obstacles and opportunities: a qualitative study of the experiences of abortion Centre staff with abortion care during the first COVID-19 lockdown in Flanders, Belgium. Sex Reprod Health Matters.

[4] Aiken ARA, Guthrie KA, Schellekens M, Trussell J, Gomperts R (2018). Barriers to accessing abortion services and perspectives on using mifepristone and misoprostol at home in Great Britain. Contraception.

[5] Smith JL, Cameron S (2019). Current barriers, facilitators and future improvements to advance quality of abortion care: views of women. *BMJ sexual &amp*. Reprod Health.

[6] Jerman J, Frohwirth L, Kavanaugh ML, Blades N (2017). Barriers to abortion care and their consequences for patients traveling for services: qualitative findings from two states. Perspect Sex Reprod Health.

[7] Doran F, Nancarrow S (2015). Barriers and facilitators of access to first-trimester abortion services for women in the developed world: a systematic review. J Fam Plann Reprod Health Care.

[8] Assifi AR, Kang M, Sullivan EA, Dawson AJ (2020). Abortion care pathways and service provision for adolescents in high-income countries: a qualitative synthesis of the evidence. PLoS One.

[9] Joyce TJ, Henshaw SK, Dennis A (2009). The impact of state mandatory counseling and waiting period laws on abortion: a literature review.

[10] Janiak E, Kawachi I, Goldberg A, Gottlieb B (2014). Abortion barriers and perceptions of gestational age among women seeking abortion care in the latter half of the second trimester. Contraception.

[11] Finer LB, Frohwirth LF, Dauphinee LA, Singh S, Moore AM (2006). Timing of steps and reasons for delays in obtaining abortions in the United States. Contraception.

[12] Van de Velde S, Van Eekert N, Van Assche K (2019). Characteristics of women who present for abortion beyond the legal limit in Flanders, Belgium. Perspect Sex Reprod Health.

[13] Ostrach B (2013). “Yo No Sabía...”—Immigrant Women’s Use of National Health Systems for Reproductive and Abortion Care. J Immigr Minor Health.

[14] Baena-Díez JM, Barroso M, Cordeiro-Coelho SI, Díaz JL, Grau M (2020). Impact of COVID-19 outbreak by income: hitting hardest the most deprived. J Public Health.

[15] Bowyer RCE, Varsavsky T, Sudre CH, et al. Geo-social gradients in predicted COVID-19 prevalence and severity in Great Britain: results from 2,266,235 users of the COVID-19 Symptoms Tracker app. 2021;76:723–725 10.1136/thoraxjnl-2020-215119 PMC8223682 33376145

[16] Marchal S, Vanderkelen J, Cantillon B, et al. The Distributional Impact of the COVID-19 Shock on Household Incomes in Belgium. [preprint] 2021.

[17] Lindberg LD, VandeVusse A, Mueller J (2020). Early impacts of the COVID-19 pandemic: findings from the 2020 Guttmacher survey of reproductive health experiences.

[18] Aiken ARA, Starling JE, Gomperts R, et al. Demand for self-managed online telemedicine abortion in eight European countries during the COVID-19 pandemic: a regression discontinuity analysis. BMJ. 10.1136/bmjsrh-2020-200880. PMC7802389 33431614

[19] Porter C, Lord J, Church K. Early medical abortion using telemedicine-acceptability to patients. *medRxiv*. 2020.

[20] Gibelin K, Agostini A, Marcot M, Piclet H, Bretelle F, Miquel L (2021). COVID-19 impact in abortions’ practice, a regional French evaluation. J Gynecol Obstetr Hum Reprod.

[21] Aiken A, Lohr PA, Lord J, et al. Effectiveness, safety and acceptability of no-test medical abortion provided via telemedicine: a national cohort study. *medRxiv*. 2020; 2020.12.06.20244921. 10.1111/1471-0528.16668 PMC8360126 33605016

[22] Centre d’Action Laïque, deMens.nu, ULB Engagée & Universiteit Hasselt. Opiniepeiling over de vrijwilligezwangerschapsafbreking in België. 2018. Consulted on the 18th of juni 2021. Retrieved from https://demens.nu/wp-content/uploads/2018/04/IVG-sondage-NL_MD.pdf

[23] Nationale Evaluatiecommissie Zwangerschapsafbreking (National Evaluation Committee on Pregnancy Termination). Nationale commissie voor de evaluatie van de wet van 3 april 1990 betreffende de zwangerschapsafbreking (wet van 13 augustus 1990) Verslag ten behoeve van het parlement 1 januari 2016–31 december 2017. 2020. Consulted on the 5th of February 2021. Retrieved from:https://overlegorganen.gezondheid.belgie.be/sites/default/files/documents/volledig_verslag_2018_nl_2020_1.pdf

[24] Döring N (2020). How is the COVID-19 pandemic affecting our sexualities? An overview of the current media narratives and research hypotheses. Arch Sex Behav.

[25] Reyniers T, Rotsaert A, Thunissen E, et al. Reduced sexual contacts with non-steady partners and less PrEP use among MSM in Belgium during the first weeks of the COVID-19 lockdown: results of an online survey. Sex Trans Infect 2020:sextrans-2020-054756. 10.1136/sextrans-2020-054756 10.1136/sextrans-2020-054756 PMC7656903 33172917

