# Supplementary material for: Abortion care in a pandemic: an analysis of the number and social profile of people requesting and receiving abortion care during the first COVID-19 lockdown (March 16 to June 14, 2020) in Flanders, Belgium
Source: Arch Public Health. 2021 Aug 4;79:140. doi: 10.1186/s13690-021-00665-6 (PMC8331997; doi:10.1186/s13690-021-00665-6)
Supplement: Supplementary file 1 — Additional file 1: Table A: Descriptive results of the continuous profile characteristics of the clients of an abortion centre in a central city in Flanders (Belgium), for the first COVID-19 lockdown (2020) and each year in the reference period (2015-2019), n = 4243. Table B: Descriptive results of the categorical profile characteristics of the clients of an abortion centre in a central city in Flanders (Belgium), for the first COVID-19 lockdown (2020) and each year in the reference period (2015-2019), n = 4243. Table C: Descriptive results of the amount of actual abortions and placing of LARC's, performed for the clients of an abortion centre in a central city in Flanders (Belgium), for the first COVID-19 lockdown (2020) and each year in the reference period (2015-2019). [file 13690_2021_665_MOESM1_ESM.docx]

**Abortion care in a pandemic: An analysis of the number and social profile of people requesting and receiving abortion care during the first COVID-19 lockdown (March 16 to June 14, 2020) in Flanders, Belgium**

**Additional files**

**Table A:** *Descriptive results of the continuous profile characteristics of the clients of an abortion centre in a central city in Flanders (Belgium), for the first COVID-19 lockdown (2020) and each year in the reference period (2015-2019), n=4243*

| Characteristic | Averages from 2015 to 2020 |
| --- | --- |
| Age | 28.58 |
|  | 28.87 |
|  | 29.12 |
|  | 29.00 |
|  | 29.53 |
|  | 29.62 |
| Number of children | 1.12 |
|  | 1.19 |
|  | 1.15 |
|  | 1.04 |
|  | 1.16 |
|  | 1.24 |
| Number of previous abortions | 0.63 |
|  | 0.64 |
|  | 0.65 |
|  | 0.67 |
|  | 0.72 |
|  | 0.72 |
| Number of miscarriages | 0.16 |
|  | 0.17 |
|  | 0.26 |
|  | 0.21 |
|  | 0.30 |
|  | 0.27 |

**Table B:** *Descriptive results of the categorical profile characteristics of the clients of an abortion centre in a central city in Flanders (Belgium), for the first COVID-19 lockdown (2020) and each year in the reference period (2015-2019), n=4243*

| Characteristics |  | % From 2015 to 2020 | Number from 2015 to 2020 |
| --- | --- | --- | --- |
| Migration background | No migration background | 45.52 | 340 |
|  |  | 44.71 | 317 |
|  |  | 43.18 | 323 |
|  |  | 43.54 | 317 |
|  |  | 40.11 | 290 |
|  |  | 40.82 | 240 |
|  | Western- and Southern Europe | 6.56 | 49 |
|  |  | 5.08 | 36 |
|  |  | 6.42 | 48 |
|  |  | 6.32 | 46 |
|  |  | 5.81 | 42 |
|  |  | 7.14 | 42 |
|  | Balkan/central Europe/western CIS and Caucasus | 13.92 | 104 |
|  |  | 11.14 | 79 |
|  |  | 9.76 | 73 |
|  |  | 11.95 | 87 |
|  |  | 14.38 | 104 |
|  |  | 11.73 | 69 |
|  | Maghreb | 8.97 | 67 |
|  |  | 10.72 | 76 |
|  |  | 12.17 | 91 |
|  |  | 11.40 | 83 |
|  |  | 11.89 | 86 |
|  |  | 11.39 | 67 |
|  | Turkey | 1.07 | 8 |
|  |  | 2.12 | 15 |
|  |  | 1.74 | 13 |
|  |  | 1.65 | 12 |
|  |  | 1.52 | 11 |
|  |  | 1.87 | 11 |
|  | Middle East | 1.20 | 9 |
|  |  | 2.54 | 18 |
|  |  | 3.48 | 26 |
|  |  | 2.34 | 17 |
|  |  | 3.18 | 23 |
|  |  | 4.59 | 27 |
|  | Sub-Saharan Africa | 10.84 | 81 |
|  |  | 12.41 | 88 |
|  |  | 12.70 | 95 |
|  |  | 12.23 | 89 |
|  |  | 11.89 | 86 |
|  |  | 11.90 | 70 |
|  | America and Oceania | 5.35 | 40 |
|  |  | 6.06 | 43 |
|  |  | 4.95 | 37 |
|  |  | 5.36 | 39 |
|  |  | 5.26 | 38 |
|  |  | 6.97 | 41 |
|  | Asia | 6.56 | 49 |
|  |  | 5.22 | 37 |
|  |  | 5.61 | 42 |
|  |  | 5.22 | 38 |
|  |  | 5.95 | 43 |
|  |  | 3.57 | 21 |
| Employment status | In paid employment | 60.91 | 455 |
|  |  | 60.23 | 427 |
|  |  | 59.49 | 445 |
|  |  | 63.60 | 463 |
|  |  | 61.13 | 442 |
|  |  | 56.46 | 332 |
|  | Studying | 7.23 | 54 |
|  |  | 6.35 | 45 |
|  |  | 7.22 | 54 |
|  |  | 7.28 | 53 |
|  |  | 6.36 | 46 |
|  |  | 7.82 | 46 |
|  | Not employed or studying | 24.10 | 180 |
|  |  | 23.41 | 166 |
|  |  | 21.26 | 159 |
|  |  | 21.43 | 156 |
|  |  | 22.13 | 160 |
|  |  | 27.21 | 160 |
|  | Unknown | 7.76 | 58 |
|  |  | 10.01 | 71 |
|  |  | 12.03 | 90 |
|  |  | 7.69 | 56 |
|  |  | 10.37 | 75 |
|  |  | 8.50 | 50 |
| Educational level | Tertiary education | 20.88 | 156 |
|  |  | 18.62 | 132 |
|  |  | 22.59 | 169 |
|  |  | 24.04 | 175 |
|  |  | 24.48 | 177 |
|  |  | 17.35 | 102 |
|  | Non-tertiary education | 62.38 | 466 |
|  |  | 58.82 | 417 |
|  |  | 59.63 | 446 |
|  |  | 66.35 | 483 |
|  |  | 65.84 | 476 |
|  |  | 48.64 | 286 |
|  | Unknown foreign education | 3.75 | 28 |
|  |  | 3.53 | 25 |
|  |  | 2.67 | 20 |
|  |  | 1.92 | 14 |
|  |  | 1.52 | 11 |
|  |  | 3.74 | 22 |
|  | Unknown | 12.99 | 97 |
|  |  | 19.04 | 135 |
|  |  | 15.11 | 113 |
|  |  | 7.69 | 56 |
|  |  | 8.16 | 59 |
|  |  | 30.27 | 178 |
| Marital status | Single/living alone | 45.92 | 343 |
|  |  | 48.24 | 342 |
|  |  | 50.53 | 378 |
|  |  | 53.16 | 387 |
|  |  | 50.07 | 362 |
|  |  | 51.36 | 302 |
|  | Married | 20.35 | 153 |
|  |  | 19.75 | 140 |
|  |  | 17.65 | 132 |
|  |  | 17.17 | 125 |
|  |  | 18.81 | 136 |
|  |  | 22.45 | 132 |
|  | Unmarried cohabitation | 29.91 | 201 |
|  |  | 24.54 | 174 |
|  |  | 26.20 | 196 |
|  |  | 24.86 | 181 |
|  |  | 25.17 | 182 |
|  |  | 22.45 | 132 |
|  | Divorced/in process/widowed | 6.83 | 51 |
|  |  | 1.48 | 53 |
|  |  | 5.61 | 42 |
|  |  | 4.81 | 35 |
|  |  | 5.95 | 43 |
|  |  | 3.74 | 22 |
| Pregnancy term | 0-8 weeks | 55.15 | 412 |
|  |  | 59.10 | 419 |
|  |  | 59.63 | 446 |
|  |  | 57.01 | 415 |
|  |  | 59.20 | 428 |
|  |  | 62.41 | 367 |
|  | 9-14 weeks | 27.98 | 209 |
|  |  | 24.82 | 176 |
|  |  | 24.60 | 184 |
|  |  | 27.61 | 201 |
|  |  | 24.76 | 179 |
|  |  | 21.77 | 128 |
|  | No abortion | 16.87 | 126 |
|  |  | 16.08 | 114 |
|  |  | 15.78 | 118 |
|  |  | 15.38 | 112 |
|  |  | 16.04 | 116 |
|  |  | 15.82 | 93 |
| Contraceptive method | No modern method | 51.14 | 382 |
|  |  | 49.37 | 350 |
|  |  | 48.26 | 361 |
|  |  | 49.04 | 357 |
|  |  | 55.33 | 400 |
|  |  | 63.78 | 375 |
|  | Modern method | 48.86 | 365 |
|  |  | 50.63 | 359 |
|  |  | 51.74 | 387 |
|  |  | 50.96 | 371 |
|  |  | 44.67 | 323 |
|  |  | 36.22 | 213 |
| Contraceptive use | No usage | 45.52 | 340 |
|  |  | 44.15 | 313 |
|  |  | 45.32 | 339 |
|  |  | 44.92 | 327 |
|  |  | 47.72 | 345 |
|  |  | 57.14 | 336 |
|  | Irregular or inaccurate usage | 30.12 | 225 |
|  |  | 30.75 | 218 |
|  |  | 29.68 | 222 |
|  |  | 32.97 | 240 |
|  |  | 29.32 | 212 |
|  |  | 26.02 | 153 |
|  | Correct usage | 19.81 | 148 |
|  |  | 18.48 | 131 |
|  |  | 20.32 | 152 |
|  |  | 14.29 | 104 |
|  |  | 16.74 | 121 |
|  |  | 12.76 | 75 |
|  | Unknown | 4.55 | 34 |
|  |  | 6.63 | 47 |
|  |  | 4.68 | 35 |
|  |  | 7.83 | 57 |
|  |  | 6.22 | 45 |
|  |  | 4.08 | 24 |

**Table C:** *Descriptive results of the amount of actual abortions and placing of LARC's, performed for the clients of an abortion centre in a central city in Flanders (Belgium), for the first COVID-19 lockdown (2020) and each year in the reference period (2015-2019)*

| Characteristics |  | % From 2015 to 2020 | Number from 2015 to 2020 |
| --- | --- | --- | --- |
| Abortion after request *(n=4243)* | No abortion | 16.78 | 125 |
|  |  | 16.13 | 114 |
|  |  | 15.89 | 119 |
|  |  | 15.60 | 112 |
|  |  | 16.28 | 117 |
|  |  | 15.94 | 93 |
|  | Abortion | 83.22 | 622 |
|  |  | 83.87 | 595 |
|  |  | 84.11 | 629 |
|  |  | 84.40 | 616 |
|  |  | 83.72 | 606 |
|  |  | 84.06 | 495 |
| Long-acting contraceptive device placed after abortion procedure (for those who received a curettage) *(n= 3096)* | Yes | 34.79 | 191 |
|  |  | 37.57 | 188 |
|  |  | 35.23 | 186 |
|  |  | 36.78 | 191 |
|  |  | 32.05 | 166 |
|  |  | 36.53 | 178 |
|  | No | 65.21 | 355 |
|  |  | 62.43 | 312 |
|  |  | 64.77 | 341 |
|  |  | 63.22 | 330 |
|  |  | 67.95 | 351 |
|  |  | 63.47 | 306 |
